# A Literature Review and Two Case Reports: Is Traumatic Dislocation of the Testes a Surgical Emergency?

**DOI:** 10.7759/cureus.24672

**Published:** 2022-05-02

**Authors:** Ahmed Al Saeedi, Ibrahim A Khalil, Abdelfttah Omran, AbdulKader Alobaidy, Abdulla Al Ansari

**Affiliations:** 1 Department of Urology, Hamad Medical Corporation, Doha, QAT

**Keywords:** testicular rupture, urological trauma, scrotal trauma, trauma, testicular dislocation

## Abstract

Traumatic dislocation of the testes (TDT) is a rare sequela of blunt scrotal and perineal trauma. TDT can easily be overlooked during concomitant trauma due to other injuries, The damage to the testis appears to be not severe in dislocation and may be corrected by repositioning even if it is delayed. However, delayed intervention might be associated with pain and discomfort and may lead to abnormal sperm parameters and possible infertility. The urgency of surgical intervention increases whenever there is associated testicular torsion, rupture, or bilateral testicular TDT. We report two cases of unilateral traumatic testicular dislocation following motorcycle crashes with different presentations and approaches to treatment. We also engage in a review of the relevant literature.

## Introduction

Traumatic dislocation of the testes (TDT) involves the displacement of one or both testes out of the scrotum [1]. It is usually unilateral, but 30% of the cases involve bilateral TDT [2]. Most published cases of TDT are due to injury from motorcycle crashes where the testes had become compressed between the fuel tank and the pubic bone. According to Raykar et al., the first treated TDT case was managed with a manual reduction in 1861, and the first surgical reduction was carried out in 1899 [3]. Since then, TDT has been reported sporadically in the literature. Prompt diagnosis and management are essential, given that the delayed treatment of TDT is associated with decreased testicular functions, including spermatogenesis [4]. We report two cases of TDT in which delayed and immediate surgical interventions had no effect on the outcomes. We also present a literature review.

## Case presentation

Case 1

**Figure 1:**
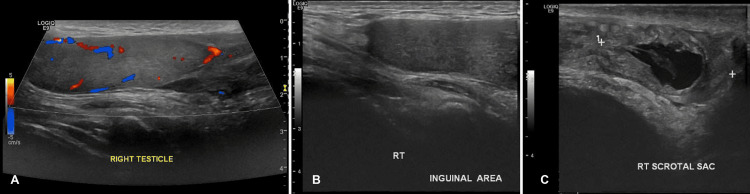
Preoperative Doppler ultrasound - case 1 The images show the right testis (panel A) in the inguinal canal (panel B) with good vascularity. Panel C depicts the scrotal sac

A 25-year-old man with no children presented to the emergency department (ED) after a motorcycle crash injury to the perineum and scrotal area. He reported concerns about right scrotal pain. He had no previous history of similar pain or abnormal testicular positioning. On physical examination, we noted an empty right scrotum with ecchymosis and swelling associated with tenderness in the right inguinal area. The Doppler ultrasound (US) confirmed the diagnosis of TDT as the right testis was in the right inguinal canal with good vascularity without ultrasonic features of tunica albuginea rupture. The testis volume was approximately 18.1 mL. The patient declined to undergo immediate surgical treatment (Figure 1).

Three months after the crash, the patient reported persistent inguinal pain. On examination, we noted that his right testis was palpable at the right inguinal area with mild tenderness. The US revealed the right testis in the right inguinal area, measuring 18 mL in volume. We proceeded with inguinal exploration and orchiopexy. Intraoperatively, we found a viable testis in the right inguinal canal with tunica albuginea tear, which was repaired, and the testis was fixed in the scrotum. Three months postoperatively, the patient's pain improved markedly. On the follow-up US, we noted normal right testis echogenicity and vascularity with a volume of 20.4 mL.

Case 2

**Figure 2:**
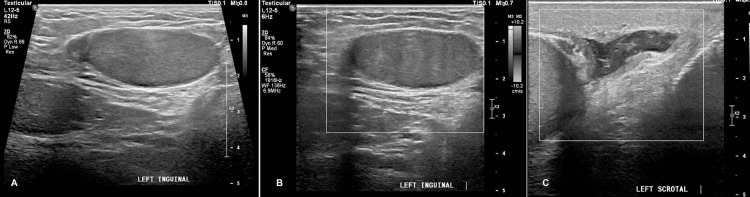
Preoperative Doppler ultrasound - case 2 The images show the left testis in the left inguinal canal (panels A, B) with no vascularity. Panel C depicts the scrotal sac

A 38-year-old man was brought to the trauma ED after being hit by a car. The patient had no history of undescended testes, and he was married with two children. We could not perform a scrotal examination initially due to the patient experiencing severe pain. He was fully conscious and oriented despite the pain. A CT scan showed multiple pelvic and spine fractures with his left testis seen at the left inguinal canal with surrounding fat stranding. A scrotal examination revealed an empty left scrotum; the severely tender testis was palpable at the external inguinal ring. The right testis was in the scrotum. The Doppler US of testes showed the left testis at the inguinal canal, relatively smaller with diffuse hypoechogenicity and decreased vascularity (Figure 2).

The patient was immediately shifted to the operating theater for inguinal exploration. The left testis was located and appeared bluish with features suggestive of testicular torsion with no tunica albuginea tear. We performed testis detorsion and orchiopexy and noted signs of viability. Three months postoperatively, the patient reported no pain, and the follow-up US revealed viable testis within the scrotum measuring 17 mL.

## Discussion

Literature review

A search of the Pubmed database was undertaken on 01/05/2022 with the search terms ((((traumatic[MeSH Terms]) OR (trauma[MeSH Terms])) AND (testis)) OR (testicular)) AND (dislocation) Non-English papers were excluded from the search. The search yielded 47 results, of which 31 papers were identified as reports on TDT in post-pubertal men [1,4-33]. A total of 33 cases of unilateral and 43 cases of bilateral TDT were reviewed. In three cases, patients had died at the scene or early after the presentation to the hospital and had been diagnosed during autopsy [5,6]. Closed manual reduction had been successful in only 16 cases [7-9]. The timing of the surgical intervention was not reported in 24 cases [7,10,11].

Blunt scrotal trauma is not an uncommon injury, especially in young men. It can lead to injuries, including minor contusion, hematoma, ruptured tunica, and shattered testis. TDT is a rare entity, and the actual incidence is difficult to determine because it is underreported. TDT is usually a consequence of high-energy trauma like motorcycle crash-related injury, although it has been reported after trivial trauma like contact with the seat of a stationary exercise bike [4]. The testis can be dislocated to different locations based on multiple factors such as the amount and direction of energy, cremasteric reflex, widely open superficial inguinal ring, an indirect hernia, and atrophic testis (Table 1) [34,35].

**Table 1:** TDT case sites in the literature TDT: traumatic dislocation of the testes

Possible locations [34,35]	Percentage [34,35]
Superficial inguinal	50
Pubic	18
Canalicular	8
Penile	8
Intra-abdominal	6
Perineal	4
Crural	2
Others	4

Diagnosis of TDT is not always straightforward and can be challenging, especially in the presence of concomitant severe injury that diverts the attention from the genital area, similar to what happened with case 2. Similarly, the presence of hematoma and inflammatory changes in the soft tissues of the scrotum can mask the diagnosis [5].

High suspicion and thorough examination are important factors in TDT diagnosis, especially during the secondary survey. The classic finding is an empty, well-developed scrotum with a palpable tender mass outside the scrotum. Doppler US is the primary investigation that can assess the viability of the testis and rule out other injuries such as hematoma, rupture, torsion, or epididymal avulsion [34,36]. A CT scan can help locate the displaced testis in difficult cases [37].

Although manual reduction is possible, it has a low success rate (15%) compared to surgical reduction; moreover, it should not be attempted when there are coexisting injuries [2,7]. Surgical intervention is the mainstay in the management of TDT. Early and delayed surgical interventions have been reported with the restoration of normal testicular function and size. The indications for immediate surgical exploration and reduction are difficulty in determining the integrity of dislocated testis, the possibility of torsion, failure of close reduction, or the minimal morbidity of an inguinal exploration [12]. A literature review of TDT cases treated with either early or delayed surgical reduction is presented in Table (2) [1,4-33].

**Table 2:** Literature review of TDT cases treated with surgical reduction and the outcome TDT: traumatic testicular dislocation; US: ultrasound

Type of dislocation	Timing of reduction	Number of cases	Outcome
Unilateral	Early	18	No atrophy, normal position, normal function, and normal US features
	Delayed	6	
Bilateral	Early	6	
	Delayed	3	

The effect of TDT on the histological features of testis has been studied by Hayami et al.; an intraoperative testicular biopsy four months after an unrepaired dislocation revealed impaired spermatogenesis but no atrophy of seminiferous tubules. A follow-up biopsy after eight months of reduction showed a slight improvement in the spermatogenesis of the testis [13]. Recovery of spermatogenesis has been reported in cases of bilateral TDT following orchiopexy even many years after the injury [5,14]. Sakamoto et al. have reported a case of an azoospermic man with four years of primary infertility and a history of TDT 15 years prior to treatment. Ten months after bilateral orchiopexy, his semen count improved, and he achieved spontaneous pregnancy with his partner 40 months after surgery [1].

We presented two TDT cases in which one received delayed surgical intervention three months following the initial injury and the other received surgical intervention immediately, and both experienced similar surgical outcomes. The main factor for immediate surgical intervention was the associated testicular torsion that necessitated the urgent intervention.

Our review and report showed that the damage to the testis does not appear to be severe in dislocation and may be corrected by repositioning even if it is delayed. However, delayed intervention might be associated with pain and discomfort and may result in abnormal sperm parameters and possible infertility. The urgency of surgical intervention increases whenever there is associated testicular torsion, rupture, or bilateral testicular TDT.

## Conclusions

TDT is a rare complication of blunt scrotal injury, and the diagnosis requires a high index of suspicion. Testicular Doppler US is the recommended imaging modality for diagnosing the condition and ruling out associated torsion or rupture. This report discussed two cases where one patient received immediate surgical intervention and the other one experienced delay in intervention for three months, yet both recovered with viable testes several months following surgical correction. Early surgical intervention is indicated, especially with associated testicular torsion or rupture of the testis, and delayed intervention is associated with pain and discomfort. Our review showed that the damage to the testis does not appear to be severe in dislocation and may be corrected by repositioning even if it is delayed.
